# Is Conduction System Pacing a Valuable Alternative to Biventricular Pacing for Cardiac Resynchronization Therapy?

**DOI:** 10.3390/jcdd11050144

**Published:** 2024-05-02

**Authors:** Davide Castagno, Francesco Zanon, Gianni Pastore, Gaetano Maria De Ferrari, Lina Marcantoni

**Affiliations:** 1 Division of Cardiology, Department of Medical Sciences, University of Turin, 10126 Turin, Italy; davide.castagno@unito.it (D.C.);; 2 Division of Cardiology, Cardiovascular and Thoracic Department, “Citta della Salute e della Scienza” Hospital, 10126 Turin, Italy; 3 Santa Maria della Misericordia Hospital, Arrhythmia and Electrophysiology Unit, 45100 Rovigo, Italy

**Keywords:** cardiac resynchronization therapy, biventricular pacing, conduction system pacing, left bundle branch area pacing, His bundle pacing

## Abstract

Cardiac resynchronization therapy (CRT) significantly improves clinical outcomes in patients with ventricular systolic dysfunction and dyssynchrony. Biventricular pacing (BVP) has a class IA recommendation for patients with symptomatic heart failure with reduced ejection fraction (HFrEF) and left bundle branch block (LBBB). However, approximately 30% of patients have a poor therapeutic response and do not achieve real clinical benefit. Pre-implant imaging, together with tailored programming and dedicated device algorithms, have been proposed as possible tools to improve success rate but have shown inconsistent results. Over the last few years, conduction system pacing (CSP) is becoming a real and attractive alternative to standard BVP as it can restore narrow QRS in patients with bundle branch block (BBB) by stimulating and recruiting the cardiac conduction system, thus ensuring true resynchronization. It includes His bundle pacing (HBP) and left bundle branch area pacing (LBBAP). Preliminary data coming from small single-center experiences are very promising and have laid the basis for currently ongoing randomized controlled trials comparing CSP with BVP. The purpose of this review is to delve into the emerging role of CSP as an alternative method of achieving CRT. After framing CSP in a historical perspective, the pathophysiological rationale and available clinical evidence will be examined, and crucial technical aspects will be discussed. Finally, evidence gaps and future perspectives on CSP as a technique of choice to deliver CRT will be summarized.

## 1. Introduction

**Figure 1:**
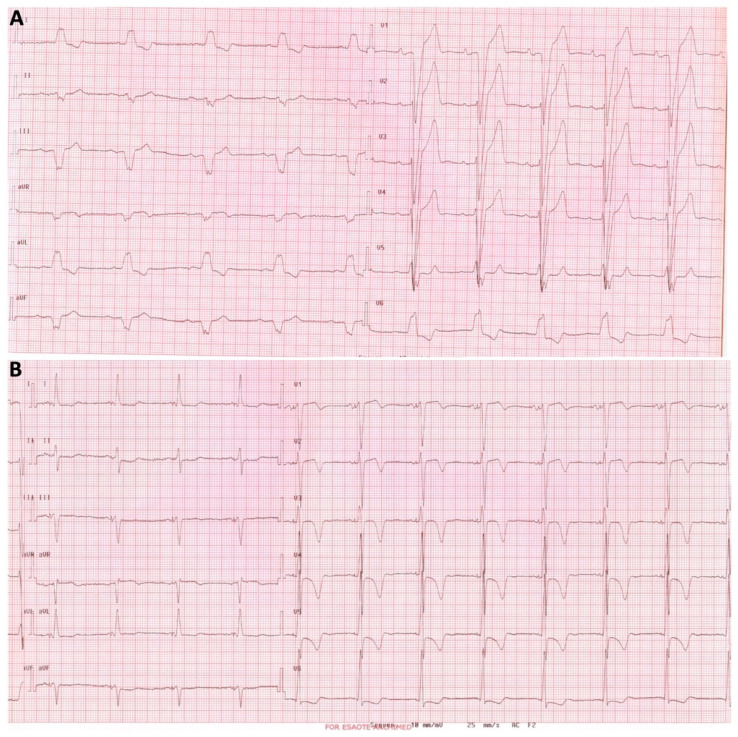
Conduction system pacing (CSP) can completely correct conduction disturbances and restore normal QRS by capturing distally to the site of the block. Panel (**A**) shows the 12-lead ECG of a patient with a paroxysmal II degree AV block and left bundle branch block (LBBB) referred for PM implantation in 2006. The His bundle was targeted, and corrective His bundle pacing (HBP) was obtained. Panel (**B**) shows paced ECG and complete QRS normalization during HBP. Of note, negative T waves in the precordial leads were a sign of cardiac memory after LBBB normalization and disappeared at the 1-month follow-up.

Biventricular pacing (BVP) is the established strategy for delivering cardiac resynchronization therapy (CRT) and is currently recommended as a first-line approach by both European and American guidelines in patients with heart failure (HF), left ventricular ejection fraction (LVEF) ≤ 35%, and wide QRS (≥130 ms) and that are symptomatic despite optimal medical therapy (New York Heart Association [NYHA] functional class II/IV) [1,2]. By inducing left ventricular (LV) reverse remodeling, BVP can significantly improve survival, reduce HF hospitalizations, and ameliorate functional capacity and quality of life. The benefit of BVP derives from the correction of inhomogeneous and delayed left ventricular (LV) electrical activation through pacing delivered from a lead in the right ventricle (RV) and a lead positioned in a branch of the coronary sinus (CS), allowing for epicardial LV stimulation. However, epicardial LV stimulation is far from physiological as electrical impulse propagates via slow cell-to-cell electrical activation with epicardial to endocardial direction; indeed, BVP can overcome LV dyssynchrony through fusion between RV paced or native endocardial activation and epicardial LV pacing. In addition, despite significant advances in delivery tools and leads, BVP is not always feasible. Difficulties in CS cannulation, lack of suitable CS tributaries, phrenic nerve capture, and high capture thresholds are some of the main pitfalls limiting successful implantation in up to 4% of cases. In some cases, a pro-arrhythmic effect of BVP has been demonstrated [3,4]. Multi-point (MPP), multi-lead (triventricular) pacing have been tested in the attempt to provide benefit when clinical improvement does not occur. Unfortunately, these strategies failed as (AV) and interventricular (VV) intervals optimization did. Therefore, even nowadays, approximately one-third of patients fail to benefit from BVP with the rate of non-responders remaining constant over time [1,5]. Given such premises, it is not surprising that alternative pacing modalities to deliver CRT have been tested. Conduction system pacing (CSP) has generated great interest in this regard as it can correct conduction disturbances and possibly restore physiological ventricular activation by capturing distally to the site of the block (Figure 1). CSP can be achieved by His bundle pacing (HBP), which is the most physiological pacing modality, or by left bundle branch area pacing (LBBAP) by engaging the LV conduction system generating a minimal delay in RV activation. CSP has been increasingly used as an alternative to BVP to deliver CRT. Several observational studies have demonstrated the feasibility and efficacy of CSP in CRT-eligible patients in improving echocardiographic outcomes and functional capacity [6].

## 2. The Rationale for Using CSP as CRT Strategy: LBBB Correction

Landmark, large, randomized clinical trials have shown that QRS duration and left bundle branch block (LBBB) morphology are strongly associated with clinically meaningful response to CRT [7,8], and current guidelines include both parameters amongst the criteria to consider when selecting patients for BVP [1,2]. A delayed LV activation pattern and the resulting inefficient myocardial mechanics make LBBB the ideal target for CRT. In this regard, the MADIT-CRT trial showed that patients with an LBBB pattern rather than non-LBBB (i.e., right bundle branch block [RBBB] or intraventricular conduction delay [IVCD]) derived significant benefit from BVP compared with an implantable cardioverter defibrillator (ICD) alone in terms of reduction of nonfatal HF events or deaths from any cause [9]. The rationale for using CSP as strategy for delivering CRT comes from the assumption that the fibers destined to become the LBB are distinct within the His bundle (HB) and can be recruited by pacing beyond the site of the block or in close proximity to it with high enough energy to overcome the conduction block. This mechanism, known as the longitudinal dissociation theory, has its anatomical basis on early histological studies by James and Sherf, who demonstrated that the HB comprised multiple bundles separated by thin collagen septa [10]. Subsequent observations in animal models [11] and preliminary clinical experiences [12] demonstrated the feasibility of QRS normalization by HB stimulation. However, restoration of normal conduction is not achievable in all LBBB patients, and the possibility to correct LBBB with HBP or LBBAP depends on the site of the conduction block. A preliminary study evaluated the LV activation pattern using electroanatomical mapping (EAM) in 10 patients with LBBB referred for BVP. The majority (71%) of patients with non-ischemic etiology showed a line of functional conduction block causing LBBB and responded to conventional BVP. In contrast, patients with a myocardial scar and homogenously slow conduction pattern from the LV septum to the lateral wall without a distinct line of conduction block often required endocardial or multisite pacing to achieve response [13]. Up to one third of patients showing an LBBB pattern have intact His-Purkinje activation, suggesting a more distal diffuse conduction disease [10]. Indeed, detection of an LBBB pattern on surface ECG could imply different disorders, such as a complete conduction block, either intra-Hisian or located at the proximal LBB; intact His-Purkinje activation; or coexistent proximal block and distal conduction delay [14], and it does not reliably predict if CSP can successfully correct the conduction disorder [15,16]. Upadhyay et al., using intracardiac linear multielectrode mapping catheters, assessed left-sided septal electrical activation among 72 patients with LBBBs [14]. They found that 36% of patients with LBBB patterns on surface ECG had preserved conduction through the His-Purkinje system, while the remaining 64% had complete conduction block at the level of the HB or LBB (44% and 18%, respectively). These findings highlight that patients with complete conduction block are potentially amenable to corrective HBP, either directly or indirectly via the “virtual cathode”. In contrast, those with intact Purkinje activation (and without complete conduction block) do not restore normal conduction during CSP, most likely because of more peripheral conduction slowing secondary to fibrosis, hypertrophy, or both [14]. Moreover, when the site of conduction block is nodal or high infranodal, HBP may represent the ideal solution, provided that good electrical parameters are achieved. When a conduction block occurs at the level of the proximal left bundle branch, LBBAP can more easily correct the underlying conduction disturbance. In the case of non-specific intraventricular conduction delay, CSP is unlikely to correct the conduction disturbance because the Purkinje system activation is intact and left ventricular activation time (LVAT) may be prolonged because of intramyocardial disease. In these circumstances, or in the case of multiple sites of blocks, hybrid approaches combining CSP and pacing through a lead placed into the CS (i.e., HOT-CRT [His-optimized CRT] and LOT-CRT [left bundle branch optimized CRT]) may be of help. When spontaneous conduction is maintained through the RBBB, fusion between the directly paced LBB and the intrinsic conduction through the right branch can generate the maximal electrical resynchronization and determine significant QRS narrowing.

## 3. Dyssynchrony

**Figure 2:**
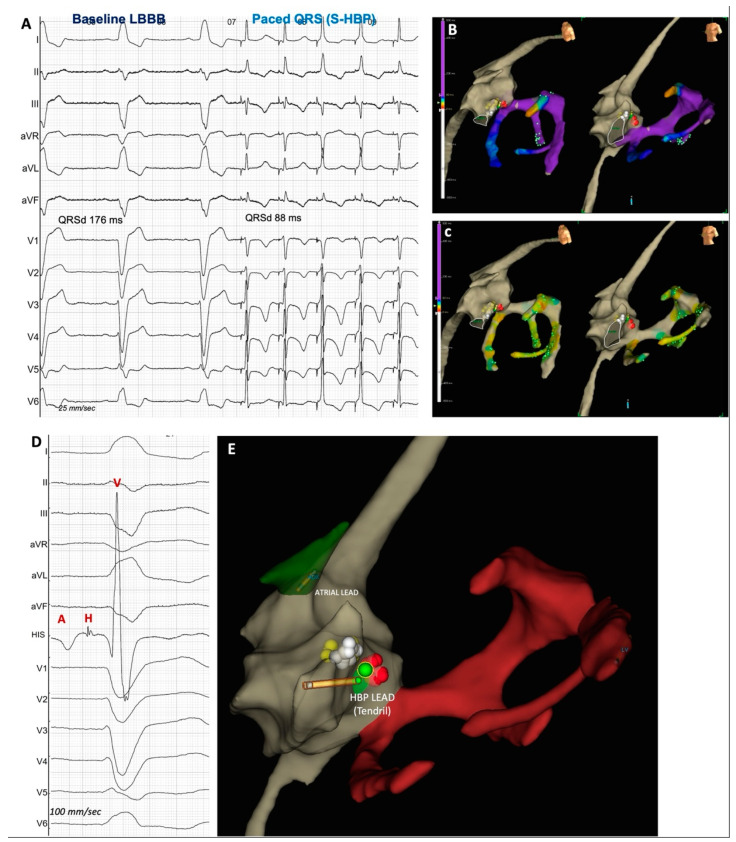
HBP that captures the His-Purkinje system has the potential to restore normal physiological activation. The depolarization front produced by His capture spreads to the ventricles by the His-Purkinje system with a faster conduction speed than through the myocardium. The figure shows the case of a patient with LBBB and severe left ventricular disfunction with CRT indication. The HBP was targeted, and complete QRS normalization was obtained. Panel (**A**) shows the spontaneous ECG (first three beats); complete LBBB and QRS duration 176 ms, followed by selective HBP-paced QRS (last five beats), with normalization of the QRS morphology and duration (88 ms). Panel (**B**,**C**) show the 3D electroanatomical reconstruction of the His cloud and the left ventricular activation mapped through a BMW guidewire inserted in all CS branches. The color code is clarified in the left margin: purple identifies late activation, while red identifies early activation. During spontaneous LBBB, the LV activation is completely delayed, with additional intraventricular delay showed by the green dot at the sparkle map appearing at different times between different CS branches. During selective HBP with QRS normalization, LV activation is equally distributed in green-yellow colors, with the green dot contemporarily appearing in all CS branches. Panel (**D**) shows the 12-lead ECG at baseline (LBBB) and the unipolar signal recorded from the His lead (“HIS”), confirming a distal position, where a small atrial signal “A”, sharp His potential “H” and big ventricular signal “V” are recorded. Panel (**E**) shows the His cloud (yellow, white, and red tags highlight, respectively, the proximal, median, and distal portion of the His Bundle). The green dot shows the exact final position of the HBP lead (Tendril lead in this case). See Supplementary Materials for the sparkle maps video.

**Figure 3:**
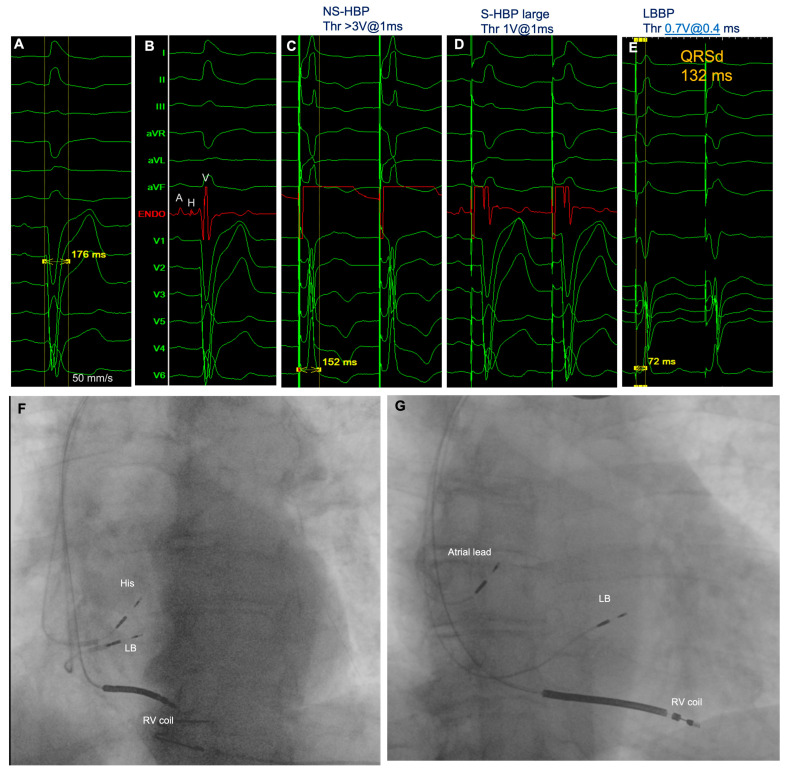
Conduction system pacing applied in a patient with LBBB and LV ejection fraction 34% as a CRT strategy. Panel (**A**): baseline spontaneous ECG (recorded at 50 mm/s speed) shows an LBBB pattern and QRSd of 176 ms. Panel (**B**): “ENDO” (red trace) represents the EGM recorded in unipolar fashion from the tip of the lead on the His Bundle (lead “His” in panel (**F**)). A: Atrial signal; H: His signal; V: ventricular signal. Panel (**C**): pacing from the His bundle produced a non-selective capture with LBBB correction at high output (threshold for LBBB correction 3 V@1 ms). A decreasing output (panel (**D**)) selective capture without LBBB correction was obtained. Considering the high output needed to correct LBBB, an additional lead was inserted to test the intraseptal left bundle branch capture and paced QRS duration, aiming to leave in place the lead with better electrical parameters and which was associated with shorter paced QRS duration. Panel (**E**) shows the paced QRS determined by the intraseptal LBB lead: the capture threshold was optimal (0.7 V@0.4 ms), left ventricular activation time (LVAT) was 72 ms, and final paced QRS duration was 132 ms. Panel (**F**) is the left anterior oblique view of the three leads: the RV coil (apical septum); “His”, the first lead implanted to test His position, thereafter moved to the atrium; and “LB”, the second lead, implanted while aiming at intraseptal LBBB pacing. Panel (**G**) shows the right anterior oblique view and the final position of the three leads: the RV coil in the apical septum, LB in LBB, and atrial lead after moving the lead from His to the atrium.

LBBB results in the delayed and dyssynchronous activation of the LV, which is deleterious in patients with HF. BVP can improve this pathological activation pattern by shortening the LVAT and enhancing cardiac function. For a long time, BVP was the only feasible option to achieve CRT. However, despite the established benefits on morbidity and mortality, BVP relies on the fusion of two ventricular activation wavefronts (i.e., one epicardial and one endocardial), prompting a non-physiologic restoration of cardiac electrical and mechanical synchrony. By directly capturing the His-Purkinje system, CSP has the potential to restore ventricular physiological activation, resulting in a more efficient electrical resynchronization compared with BVP, as demonstrated by a greater reduction in LVAT and more pronounced hemodynamic improvements [17]. The depolarization front produced by His capture spreads across the ventricles via the His-Purkinje system, with a faster conduction speed than the cell-to-cell method through the myocardium (Figure 2). This true resynchronization can lead to the recovery of LV mechanical synchrony and reverse remodeling with consequent improvement in LVEF. Due to the inherent challenges and limitations of HBP, LBBAP has most recently been attracting increasing interest. Indeed, LBBAP offers several technical advantages compared with HBP (e.g., higher implant success rate, lower capture threshold, larger sensed R wave amplitude) (Figure 3). Furthermore, full LBBB correction is more often and more easily achieved with a subsequent higher rate of reverse remodeling and better clinical outcomes [18]. According to the recent prospective randomized study LBBP-RESYNC, LBBAP demonstrated greater LVEF improvement and greater left ventricular end-systolic volume reduction than BVP in 40 patients with non-ischemic cardiomyopathy and LBBB followed over 6 months [19]. A potential disadvantage is that the RV activation does not occur via the conduction system. In fact, during LBBAP, the ventricles are activated by two different wavefronts: one from the conduction system and one from the surrounding ventricular myocardium.

## 4. Hemodynamics

Hemodynamic studies have been used to compare CSP with BVP and to evaluate the acute impact of these pacing modalities on cardiac function. In 2016, Padeletti et al. [20], in a small prospective study (11 patients enrolled), showed enhanced systolic function and LV synchrony when LV epicardial pacing was combined with HBP, regardless of the atrioventricular delay settings. Based on these findings, the authors suggested that sequential HBP-LV activation can provide significant hemodynamic benefits by preserving intrinsic RV activation. More recently, a prospective study evaluated the acute electrophysiological and hemodynamic effects of BVP compared with temporary HBP and LV septal (LVS) pacing obtained by a quadripolar lead positioned at the level of the left ventricular endocardial side using a retrograde aortic approach. Both HBP and LVS pacing provided similar electrical resynchronization (i.e., QRS area and activation times reduction) and had superior results than BVP. The short-term hemodynamic effect measured by dP/dt max was similar for all three pacing configurations. Interestingly, the effects of LVS pacing were independent of the septal pacing location (i.e., basal, mid, apical septum) [21]. A within-patient comparison of the effects of HBP and BVP on ventricular activation time (measured using ECG imaging) and acute hemodynamic function was performed in patients with HF and LBBB. In 18 out of 23 patients, LVAT was significantly shortened by HBP, which produced more effective ventricular resynchronization than BVP (LVAT −26 ms; 95%CI: −41 to −21 ms; *p* 0.002). This translated into a greater improvement in hemodynamic response, with a 60% increase in acute systolic blood pressure compared with BVP (+4.6 mmHg; 95% CI: 0.2–9.1 mmHg; *p* 0.04). These findings suggest that, whenever HBP can successfully correct LBBB, it has the potential to deliver more effective ventricular resynchronization, ultimately improving cardiac function [17]. Whenever HBP fails to shorten QRS duration, HOT-CRT may offer a valuable alternative. In 19 candidates to CRT, because of LV impairment and concomitant conduction abnormalities, HOT-CRT produced a 24% greater reduction in LVAT compared with BVP (LVAT −22 ms; 95% CI: −33 to −10 ms; *p* 0.002) [22]. The acute hemodynamic effects of LBBAP versus BVP in patients with LBBB and concomitant LV impairment mainly of non-ischemic etiology were recently investigated. LBBAP produced a significantly greater reduction in QRS duration compared with BVP (−11 ms; 95% CI: −17 to −4 ms; *p* 0.003) and a greater reduction in QRS area (−85 μVs [95% CI, −113 to −56 μVs]; *p* < 0.001). These positive effects on ventricular resynchronization translated into a greater acute increase in LV dP/dt for LBBAP compared with BVP (6% [95% CI, 2%–9%]; *p* = 0.002) [23]. The LEVEL-AT (left ventricular activation time shortening with conduction system pacing vs. biventricular resynchronization therapy) study randomized 70 patients to CSP vs. BVP with the primary endpoint being the change in LVAT measured using electrocardiographic imaging (ECGi) 45 days post-implantation. In the intention-to-treat analysis, CSP resulted non-inferior to BVP in reducing LVAT (LVAT CSP: −28 ± 26 ms vs. BVP: −21 ± 20 ms; *p* < 0.001 for non-inferiority) [24]. In order to investigate whether RV delayed activation adversely impacted cardiac function, Ali et al. performed a within-patient acute hemodynamic study comparing HBP and LBBAP in 19 patients with LBBB and LV dysfunction. Noninvasive electrical mapping confirmed that the delayed RV activation with LBBAP did not adversely affect hemodynamic response (*p* 0.8); LBBAP was not inferior to HBP in reducing the LVAT (*p* 0.65). The HBP produced more rapid biventricular activation compared with LBBAP (P 0.03) [25]. 

## 5. CSP to Achieve CRT: Preliminary Experiences

HBP was the first pacing strategy tested to correct LBBB. The concept of functional longitudinal dissociation within the HB [10,11,12] provides the anatomical and pathophysiological foundation to the observation that, in some patients with LBBB, QRS narrowing and concomitant normalization of ventricular electromechanical synchrony can be achieved during HBP [11]. In the 1970s, Narula [12] firstly reported QRS normalization in 25 patients with LBBB advancing within the HB and pacing distally to the presumed site of block, thus suggesting that a block within the HB could be bypassed [12]. In patients with acute RBBB after myocardial infarction and patients with chronic LBBB, El-Sharif and colleagues [11] were able to normalize QRS with distal HBP. Furthermore, they demonstrated that in canine models where the septal artery was ligated, resulting in intra-hisian delay and BBB, distal pacing resulted in the normalization of the QRS in two-thirds of cases. In 2000, Deshmukh et al. [26] first described permanent HBP in twelve patients with chronic AF and LV systolic dysfunction undergoing AV node ablation. Clear beneficial effects, including reduction in LV end-diastolic and end-systolic diameters and increase in LVEF and fractional shortening were observed at follow-up. Since then, several studies have demonstrated the feasibility of permanent HBP and potential associated benefits compared with conventional RV pacing, including improvements in functional capacity, ventricular synchrony, and LVEF. In 2013, Barba-Pichardo et al. published the first experience of LBBB correction by permanent HBP [27]. Subsequently, Lustgarten presented the first crossover study on HBP compared with BVP in patients with HF and reduced LVEF. Both the HBP and CS lead were connected to the LV port by a Y-adapter to enable either pacing modality. Patients were randomized to HBP vs. BVP and, after 6 months, crossed over to the other pacing modality for another 6-month period. The feasibility of HBP, together with the possibility to re-engage preserved left fascicular tissue with QRS normalization were demonstrated in 72% of patients (mean QRS duration: baseline 171 + 13 ms; HBP 148 + 11 ms; BVP 158 + 21 ms, *p* < 0.0001). Clinical and echocardiographic responses were similar in HBP compared with BVP, with similar improvement in baseline LVEF from 26% to 32% (in HBP) and 31% (in BVP) at 6 months, suggesting that HBP was as efficient as BVP in this cohort [28]. Another study by a Spanish group published in 2011 showed how HBP used as bail out strategy whenever standard BVP via the CS was unsuccessful, in addition to electrical resynchronization and optimal clinical response, induced ventricular mechanical synchrony. Immediate abolition of septal-to-posterior wall delay and the disappearance of basal conduction disturbances due to LBBB were evident at the M-mode color tissue Doppler echocardiography during HBP; pulsed-wave tissue Doppler demonstrated the shortening of isovolumetric conduction time with higher peak systolic velocity whilst narrowing QRS by HBP; LVEF and LV diameters significantly improved [29]. Padeletti et al. tested simultaneous temporary HBP and LV epicardial pacing, showing an improvement in systolic function at hemodynamic evaluation beyond standard BVP regardless of the atrioventricular interval setting. The authors explained these findings, suggesting that HBP does not deliver an additional electric wave front to the right ventricle; instead, it takes advantage of the right bundle branch conduction (which is usually preserved in LBBB) and enables fusion between the LV pacing induced wavefront and intrinsic conduction [20]. More recently, Ajijola et al. tested the feasibility of HBP in lieu of an LV lead, obtaining a successful implant in 76% of cases (16 of the 21 patients enrolled). HBP induced a significant improvement in LVEF and functional class [30]. Starting from these preliminary experiences, several studies have been conducted to test CSP as an alternative to BVP in HFrEF patients, both in BVP non-responders and as a primary strategy. A recent comprehensive systematic review analyzed electrocardiographic, echocardiographic, and clinical outcomes after CSP compared with BVP in patients with CRT indications. Pooling the results from 6 randomized controlled studies and 12 observational nonrandomized studies, this meta-analysis showed the advantages of CSP in terms of QRS shortening, left ventricular remodeling, survival, and heart failure decompensation as compared with BVP [31].

## 6. Technical Considerations

Despite several investigations proving the feasibility and efficacy of HBP as CRT strategy, technical challenges associated with this pacing methodology had been an obstacle to its routine application. More in detail, the perceived lower implant success rate and higher pacing threshold associated with HBP are potential limitations to this approach. In contrast, LBBAP is associated with a low and stable capture threshold, optimal sensing, and higher procedural success rate thanks to the larger target area and smaller amount of surrounding fibrous tissue. This makes LBBAP suitable for sensing ventricular arrhythmias in addition to the aforementioned resynchronization capabilities. This possibility was tested in the CROSS-LEFT pilot study [32], which enrolled 10 patients with reduced ejection fraction and complete LBBB, who received a DF-1 dual chamber ICD with a single LBBAP lead connected to the IS-1 port; the defibrillator lead was positioned at the right ventricular apex to the DF-1 port of the device. At the time of implantation, ventricular fibrillation was induced, and both conventional (apical) and left bundle branch area sensing configurations were tested. No significant sensing differences were observed, but LBBAP was associated with electromechanical reverse remodeling and the improvement of LVEF at 6 months follow-up [32]. More recently, another study with a slightly bigger sample size (*n* = 30), assessed R-wave sensing and the long-term reliability of the LBBAP lead for the appropriate detection of ventricular arrhythmias in patients requiring CRT with defibrillator therapy [30]. During a mean follow-up of 23 months, ventricular arrhythmias detection from LLBAP lead was overall safe (i.e., 89% of episodes were appropriately detected), with 11% of episodes being inappropriately detected because of T wave oversensing in a single patient [30]. It should be noted that the adoption of LBBAP as a CRT strategy in ICD recipients requires the presence of multiple leads in the RV, possibly causing a lead-to-lead interaction, which can damage insulation and conductors and can increase the degree of tricuspid regurgitation. During implantation, great care should be taken to maintain adequate distance between the defibrillator coil and the ring of the LBBAP lead. Indeed, repetitive lead–lead interaction, especially when the defibrillator coil slides against the ring of the LBBAP lead, can determine insulation breach and damage the conductor, possibly causing lead fracture in the place where the lead is in contact with the coil [33]. This mechanism was clearly elucidated with bench tests reproducing fracture of the conduction cables within polyurethane-insulated leads [34]. Furthermore, noise-like signals on the LV channel caused by intermittent contact between the RV coil and the ring electrode of the LBBAP lead can lead to oversensing and inappropriate pacing inhibition from the LBBAP [35]. Whenever an LBBAP lead is added to a defibrillator lead, it is mandatory to check the leads’ position from multiple fluoroscopic views to avoid lead-to-lead interaction. This phenomenon may also be prevented by careful evaluation of both the bipolar (tip-to-ring) and the unipolar (ring-to-skin) signals during implantation. A first-in-human feasibility study with an ICD lead positioned at the level of the left bundle branch area was recently reported [36]. The implantation success rate was 60% (3 out of 5 patients), and the mean procedural and fluoroscopy duration were 170.0 ± 17.3 min and 28.8 ± 16.1 min, respectively [37]. In the near future, the availability of a dedicated LBBAP-defibrillator lead could make CSP and tachyarrhythmias detection/treatment with a single lead possible. CSP management during follow-up could be challenging. CSP leads generally pace multiple structures simultaneously (i.e., conduction system and surrounding myocardium) with or without the correction of the conduction block. Each tissue will show its own threshold, which should be identified to properly tailor output aiming to correct the bundle branch block. Furthermore, until now, available devices did not specifically address CSP programming requirements. Just recently, dedicated full systems (device and lead) for LBBAP have received CE approval.

## 7. Procedural Success Rate

Using CSP as strategy to achieve CRT does not guarantee a 100% implant success rate, as shown in both retrospective observational studies and in prospective randomized trials. His-SYNC and His-alternative randomized trials compared HBP with BVP. The HBP was equivalent or even superior to BVP in terms of QRS narrowing and echocardiographic response. However, crossover to BVP was required in a high proportion of patients randomized to HBP because of implantation failure. Moreover, in the His-SYNC pilot study, the high crossover rate was ascribed to the inclusion of patients with IVCD, not amenable to HBP-mediated QRS correction and reverse remodeling [36]. Unsurprisingly, in the His-alternative study, which excluded patients with IVCD, crossovers were less frequent, and the overall procedural success was higher [38]. Implantation success rates are usually satisfying with LBBAP, reaching >90% in the context of conventional bradyarrhythmic indications and >80% for HF indications, according to the MELOS study [39]. However, similarly to traditional BVP, the presence of a septal scar could be associated with implant failure. In a retrospective single-center study of 25 ischemic cardiomyopathy patients, LBBAP was unsuccessful in 36% of patients, showing a high scar burden on cardiac magnetic resonance and requiring BVP as alternative pacing modality to achieve CRT [40]. The presence of fibrotic tissue at the level of the mid-basal interventricular septum can hamper lead penetration and prevent the capture of the conduction system in the proximity of the LV subendocardium.

## 8. Complications

In a recent meta-analysis comparing CSP and BVP in CRT-eligible patients, the overall complications and lead revision rates were not significantly different between the two pacing modalities (6% in the CSP group vs. 8% in the BVP group, OR 0.80, 95% CI: 0.53–1.23). The rates of lead revision were also similar (OR 0.74, 95% CI:0.51–1.08). In addition, no significant differences were found for HBP versus LBBAP [41]. These results are concordant with the observations made in the MELOS study, where 27.5% of included patients had a primary pacing indication for HF. The overall complication rate with LBBAP was approximately 8%, including 4% of acute septal perforations, which usually do not have clinically relevant sequela following the intervention [39]. In another meta-analysis comparing LBBAP and BVP, the pacing threshold was lower in the former group, which was also associated with shorter QRS duration. Greater LVEF improvement, NYHA class, and lower risk of HF hospitalizations were also observed in patients receiving LBBAP compared with BVP [42]. Although HBP is highly effective at resynchronizing the heart, the unpredictable rise in capture threshold during follow-up is one of the greatest concerns with this pacing modality, which is especially relevant to premature battery depletion and the need for lead revision. Data on the long-term follow-up of HBP derive from the observational data of patients implanted for bradyarrthymias. Lead revision was required in up to 6% of cases, mainly due to technical pitfalls at the time of implant (i.e., abnormal slack shape, non-perpendicular angle of lead insertion) [43]. Recently, few cases of lead fracture of deep intraseptal leads have been reported. This problem seems to occur with both stylet-driven and lumenless leads, but with different underlying mechanisms. Although rare (i.e., 2/325, 0.6% of implants aiming at LBBAP), fractures of stylet-driven leads seem to occur relatively early during follow-up (median follow-up 18 months), with the conductor between the tip and ring of the electrode being involved [44]. A fractured lumenless lead proximally to the ring electrode was recently reported 2 years after a challenging implant (four tested positions), suggesting that the number of deployment attempts may be associated with a higher risk of fracture [45]. Altogether, the risk of lead fracture could be related to the repetitive mechanical stress imposed on deep intraseptal leads as well as to the possibility of lead damage resulting from multiple screwing attempts during implant. In addition, excessive lead angulation and preconditioning might contribute to early lead fracture. Because of the deep septal location achieved in LBBAP, extractability of malfunctioning or infected leads raises concerns, especially in the case of leads with long-dwelling times, for which clinical experience is still limited.

## 9. Current Guidelines

The emerging role of CSP as an alternative pacing modality to achieve CRT lays its foundation on the high proportion of non-response following BVP. The recently published 2023 HRS/APHRS/LAHRS guidelines assign a class IIb recommendation for CSP (i.e., HBP or LBBAP) as an alternative to BVP in patients with LVEF < 35%, sinus rhythm, LBBB, QRS duration >150 ms, NYHA class II-IV despite guideline-directed medical therapy [2]. A class IIa recommendation for CSP is considered in case an effective CRT cannot be achieved with BVP and in patients with EF 36–50% with anticipated substantial (i.e., ≥20–40%) ventricular pacing as an alternative to BVP. Similarly, the latest ESC guidelines on cardiac pacing proved a class IIa recommendation for HBP in CRT candidates in whom CS lead implantation is unsuccessful [1].

## 10. Relevant Studies and Ongoing Trial

Observational studies and a small number of randomized controlled trials have reported a greater improvement in soft endpoint such as LVEF improvement, QRS duration, and hemodynamic parameters and quality of life with CSP compared with BVP [46,47]. In the His optimized pacing evaluated for heart failure (HOPE-HF) randomized, double-blind, crossover trial enrolling patients with HFrEF and PR interval ≥200 ms and QRS ≤ 140 ms (or bundle branch block), HBP did not increase peak oxygen uptake but improved heart failure specific quality of life compared with a strategy of no pacing [48]. In the left bundle branch pacing versus biventricular pacing for cardiac resynchronization therapy (LBBP-RESYNC) trial, the efficacy of LBBAP was compared with BVP with respect to the improvement of LVEF and other measures of cardiac function among patients with nonischemic cardiomyopathy and complete LBBB. At 6 months follow-up, a greater improvement of LVEF was observed after LBBAP vs. BVP (mean difference 5.6%, 95% CI: 0.3–10.9; *p*-value = 0.039). LBBAP recipients also showed a greater reduction in end-systolic volume and in NT-proBNP levels compared with BVP, although QRS narrowing was comparable between the two groups [19]. More recently, the feasibility and efficacy of HOT-CRT was compared with BVP in patients with heart failure, reduced LVEF (<50%), and CRT indications. A higher procedural success was observed with HOT-CRT compared with BVP (96% vs. 82%, *p*-value 0.03). At 6 months follow-up, HOT-CRT resulted in greater LVEF improvement compared with BVP (12.4% ± 7.3% vs. 8.0% ± 10.1%, *p*-value = 0.02) [49]. A recent meta-analysis compared the hard clinical outcomes of CSP versus BVP. In total, 21 studies (4 observational and 17 randomized controlled trials) were analyzed, and 1960 patients assigned to CSP and 2367 patients assigned to BVP were included. After a median follow-up of 10.1 months, CSP was associated with a significant decrease in all-cause mortality (OR 0.68; 95% CI: 0.56–0.83, I2 0%) and in the risk of HF hospitalization (OR 0.52; 95% CI: 0.44–0.63, I2 0%). LVEF improvement and NYHA class reduction were also greater in the CSP group compared with the BVP group [41]. Of note, a number of randomized controlled studies are currently ongoing and will be terminated in the next few years, possibly providing results that will help change the current approach to CRT (Table 1).

## 11. Gaps and Unmet Needs

CSP could be reasonably considered a valid alternative to conventional BVP for CRT candidates, but the lack of large, randomized studies still precludes the dissemination of this approach in daily clinical practice. Indeed, according to current guidelines [1,2,50], CSP may be offered as a bail-out option in CRT patients when CS lead implantation fails; to maintain physiological ventricular activation when a high burden of ventricular pacing is anticipated in patients with mildly reduced LVEF; or in the context of a pace and ablation strategy in patients suffering from tachycardiomyopathy. A European Heart Rhythm Association (EHRA) consensus document was recently published in an attempt to standardize CSP implantation [51]. Further improvements in implantation techniques and the development of dedicated implant tools may lead to a further increase in the procedural success rate along with a decrease in complications. Based on the mechanism of re-establishing inter- and intraventricular synchrony, BVP gained the acronym of CRT, and over time, the terms BVP and CRT have been used interchangeably. Indeed, CRT is a more comprehensive term that comprises the whole setting of simultaneous and non-simultaneous BVP stimulation as well as LV-only pacing and CSP options. Experience with CSP has been increasing; however, CSP still lacks its own terminology, possibly generating some confusion, as now the acronym CRT is no longer synonymous with BVP only but could also refer to CSP. A terminology update is expected to include the resource of CSP [52].

## 12. Conclusions

Currently, BVP is the only pacing strategy in HFrEF that has been proven effective in improving cardiac function, functional capacity, and survival. However, response to BVP is variable, ranging from complete normalization of cardiac function to lack of benefit. CSP is increasingly used as an alternative to BVP to achieve CRT. Selected patients with HFrEF might experience a positive electro-mechanical resynchronization with CSP, which has shown promising preliminary results and is currently undergoing rigorous clinical investigations.

## Figures and Tables

**Table 1:** Principal ongoing RCTs comparing CSP vs. BVP to achieve CRT.

Nct Number	Study Title	Location	Status	Study Design	Primary Outcomes	Number of Patients	Follow-up
NCT06278844	Exercise Capacity Improvement by Conduction System Pacing in heArt Failure patieNts Without Compelling CRT inDication (ESCPAND)	Belgium	Recruiting	Interventional Randomized Parallel assignment Open label	Exercise capacity (change in VO_2_ peak from baseline to 24 weeks)	75	24 weeks
NCT04409119	Direct HIS/LBB Pacing as an Alternative to Biventricular Pacing in Patients with HFrEF and a Typical LBBB (HIS-alt_2)	Denmark	Recruiting	Randomized Parallel assignment Double masking (participant, outcome assessor)	Change in Left ventricular end-systolic volume (decrease in left ventricular systolic volume of ≥15% of baseline) Success rate of implanting a HIS-bundle lead with capture of the left bundle branch or a LBB-lead with narrowing of QRS	125	6 months
NCT05650658	Left vs. Left Randomized Clinical Trial	USA	Recruiting	Randomized Parallel assignment Triple masking (participant, care provider, outcome assessor)	Combined clinical endpoint of all-cause mortality and hospitalization for heart failure	2136	5.5 years
NCT06105580	Conduction System Pacing vs. Biventricular Pacing in Systolic Dysfunction and Wide QRS: Mortality, Heart Failure Hospitalization or Cardiac Transplant (CONSYST-CRT II)	Spain	Recruiting	Interventional Randomized Parallel assignment Single masking (participant)	All-cause mortality, cardiac transplant, or heart failure hospitalization	320	12 months
NCT06241651	CSP Versus BiVP for Heart Failure Patients With RVP Upgraded to Cardiac Resynchronization Therapy: a Prospective Multicenter Non-inferiority Randomized Controlled Study (CSP-UPGRADE)	China	Recruiting	Interventional Randomized Parallel assignment Open label	ΔLVEF (change in LVEF from baseline)	66	6 months
NCT05467163	Conduction System Pacing Versus Biventricular Pacing After Atrioventricular Node Ablation (CONDUCT-AF trial)	Austria, Bulgaria, Belgium, Croatia	Recruiting	Interventional Randomized Parallel assignment Open label	Change in left ventricular ejection fraction	82	6 months
NCT05428787	Resynchronization in Patients With HF in AF Trial Undergoing Pace & AVNA Strategy With LBBAP Compared With BiV Pacing (RAFT-P&A)	Canada	Recruiting	Randomized Parallel assignment Masking double (participant, outcome assessor)	Change in NT-proBNP from baseline	284	6 months
NCT05434962	The Left Bundle Cardiac Resynchronization Therapy Trial (LEFT-BUNDLE-CRT)	Spain	Recruiting	Randomized Parallel assignment Open label Non-inferiority.	CRT response (improvement of a clinical composite score or ≥15% reduction in left ventricular end-systolic volume)	176	6 months
NCT06052475	Physiological Versus Right Ventricular Outcome Trial Evaluated for Bradycardia Treatment Upgrades (PROTECT-UP)	UK	Recruiting	Randomized Crossover assignment Masking quadruple (participant, care provider, investigator, outcome assessor)	SF-36 physical component summary	155	14 months
NCT05265520	His-Bundle Corrective Pacing in Heart Failure (HIS-CRT)	USA	Recruiting	Randomized Parallel assignment Single masking (outcome assessor)	Change in left ventricular ejection fraction in heart failure patients with Right bundle branch block (RBBB)	120	6 months
NCT05572957	LBBP as Initial Therapy in Patients With Non-ischemic Heart Failure and LBBB (LIT-HF)	China	Recruiting	Randomized Parallel assignment Open label	Proportion of patients requiring ICD implantation for prevention of sudden cardiac death after treatment with two strategies (GDMT, LBBP + GDMT), the percentages of LVEF still ≤35% and/or ventricular arrhythmia events was assessed in both groups.	50	6 months
NCT05814263	HIS Alternative II—UK Site	UK	Recruiting	Randomized Parallel assignment Double masking (participant, outcome assessor)	Change in left ventricular end-systolic volume (decrease in left ventricular systolic volume of ≥ 15% of baseline) Success rate of implanting a HIS-bundle lead with capture of the left bundle branch or a LBB-lead with narrowing of QRS The success rate of implanting a pacing lead to the HIS-bundle, with capture of the left bundle at a threshold < 2.5 V at 1 ms or implantation of a LBB lead with narrowing of the QRS duration and maintaining this effect at 6 month follow-up	40	6 months
NCT03803995	Mapping and Pacing of the His Bundle for Heart Failure Patients With Left Bundle Branch Block (MAP HIS HF)	USA	Recruiting	Single group assignment Open label	Successful HB pacing sites Collect 3D Locations and electrogram characteristics (morphology and activation time) at the sites where His bundle (HB) pacing is associated with left bundle recruitment and corrects electrical dyssynchrony at HB pacing implant procedure.	30	N/A
NCT05769036	Conventional Biventricular Versus Left Bundle Branch Pacing on Outcomes in Heart Failure Patients (RECOVER-HF)	Russia	Recruiting	Randomized Parallel assignment Open label	All-cause mortality or worsening of heart failure requiring unplanned hospitalization (%)	60	24 months
NCT05187611	Conduction System Pacing vs. Biventricular Resynchronization Therapy in Systolic Dysfunction and Wide QRS: CONSYST-CRT.	Spain	Active, not recruiting	non-inferiority trial Randomized Parallel assignment Single masking (participant)	Composite endpoint consisting of all-cause mortality, cardiac transplant, heart failure hospitalizations, and left ventricular ejection fraction (LVEF) improvement <5 points	130	1 year
NCT05572736	Conduction System Pacing Versus Biventricular Resynchronization in Patients With Chronic Heart Failure (PhysioSync-HF)	Brazil	Active, not recruiting	Interventional Randomized Parallel assignment Masking Double (participant, outcomes assessor)	Non-inferiority of clinical benefit, a hierarchical composite of all-cause death, any hospitalization for heart failure, any urgent heart failure visit, and left ventricular ejection fraction change at 12 months.	179	12 months
NCT05155865	Conduction System Pacing Versus Biventricular Pacing for Cardiac resynchronization (CSP-SYNC)	Slovenia	Active, not recruiting	Interventional Randomized Parallel assignment Open label	Change in left ventricular volume, left ventricular ejection fraction, difference in heart failure class, proBNP value, 6-min walk test distance, EQ-5D index score	62	12 months
NCT05760924	Left Bundle Branch Pacing on Outcomes and Ventricular Remodeling in Biventricular CRT Nonresponders (RESCUE)	Russia	Not yet recruiting	Randomized Parallel assignment Open label	All-cause mortality or worsening of heart failure requiring unplanned hospitalization (%)	30	24 months
NCT06207383	Atrial Fibrillation Ablation Versus Atrioventricular Nodal Ablation With Conduction System Pacing in Heart Failure (ABACUS trial)	Geneve	Not yet recruiting	Interventional Randomized Parallel assignment Masking single (outcome assessor)	Superiority endpoint: incidence of mortality and cardiovascular hospitalization in each arm Non-inferiority endpoint: incidence of mortality and heart failure hospitalization in each arm	220	1–4 years
NCT06342492	Conduction System Vs Surgical Left Ventricular Epicardial Pacing For Coronary Sinus Lead Failure (KCHRRF_CS Lead Failure_0025)	USA	Not yet recruiting	Observational Cohort Retrospective	Need for lead revision between conduction system pacing (CSP) and transthoracic left ventricular (LV) epicardial pacing approach	100	N/A
NCT05793502	Left Bundle Branch Pacing (LBBP) for the Treatment of Cardiac Dysfunction With Safety and Efficacy Study in Patients With Atrioventricular Block	China	Not yet recruiting	Prospective Case-control	Change in LVEF	160	12 months

## Data Availability

No new data were created or analyzed in this study. Data sharing is not applicable to this article.

## Supplementary Materials

The following supporting information can be downloaded at: https://www.mdpi.com/article/10.3390/jcdd11050144/s1, Video S1: RS prop 2; Video S2: His prop 3.
